# Nusinersen Improves Motor Function in Type 2 and 3 Spinal Muscular Atrophy Patients across Time

**DOI:** 10.3390/biomedicines12081782

**Published:** 2024-08-06

**Authors:** Bogdana Cavaloiu, Iulia-Elena Simina, Crisanda Vilciu, Iuliana-Anamaria Trăilă, Maria Puiu

**Affiliations:** 1 Faculty of Medicine, Department of Microscopic Morphology, Genetics Discipline, Center of Genomic Medicine, ‘Victor Babes’ University of Medicine and Pharmacy Timisoara, 2 E. Murgu, Sq., 300041 Timisoara, Romania; bogdana.cavaloiu@gmail.com; 2 Department of Radiology, ‘Victor Gomoiu’ Children’s Clinical Hospital, 21 Basarabia Blvd., 022102 Bucharest, Romania; 3 Department of Genetics, Center of Genomic Medicine, ‘Victor Babeş’ University of Medicine and Pharmacy of Timișoara, 300041 Timisoara, Romania; maria_puiu@umft.ro; 4 Department of Neurology, ‘Carol Davila’ University of Medicine and Pharmacy, 020021 Bucharest, Romania; crisanda.vilciu@umfcd.ro; 5 Neurology Clinic, ‘Fundeni’ Clinical Institute, 022328 Bucharest, Romania; 6 Department of Pathology, ‘Pius Brinzeu’ Emergency County Clinical Hospital, 300723 Timisoara, Romania; trailaiuliana@gmail.com

**Keywords:** spinal muscular atrophy, SMA, motor function, nusinersen, genetic disorder, gene expression, mutation, SMN1, risdiplam, target therapy, RULM, HFMSE

## Abstract

Spinal muscular atrophy (SMA) is a genetic disorder primarily caused by mutations in the SMN1 gene, leading to motor neuron degeneration and muscle atrophy, affecting multiple organ systems. Nusinersen treatment targets gene expression and is expected to enhance the motor function of voluntary muscles in the limbs and trunk. Motor skills can be assessed through specific scales like the Revised Upper Limb Module Scale (RULM) and Hammersmith Functional Motor Scale Expanded (HFMSE). This study aims to evaluate the influence of nusinersen on the motor skills of patients with SMA Type 2 and 3 using real-world data collected over 54 months. A prospective longitudinal study was conducted on 37 SMA patients treated with nusinersen, analyzing data with R statistical software. The outcomes revealed significant improvements in motor functions, particularly in SMA Type 3 patients with higher RULM and HFSME scores. Additionally, GEE analysis identified time, type, age, and exon deletions as essential predictors of motor score improvements. The extended observation period is both a major strength and a limitation of this research, as the dropout rates could present challenges in interpretation. Variability in responses, influenced by genetic background, SMA type, and onset age, highlights the need for personalized treatment approaches.

## 1. Introduction

Spinal muscular atrophy (SMA) is a rare autosomal recessive neurodegenerative disorder stemming from a deletion or mutation in the Survival Motor Neuron 1 SMN1 gene [1,2]. The homozygous deletion of Exon 7 or Exons 7 and 8 was observed in ~95% of SMA patients and compound heterozygous mutation in ~5% of patients [3]. This condition leads to the degeneration of α-motor neurons found in the anterior horn of the spinal cord and lower brainstem, leading to insufficient SMN protein for motor neuron health [4,5,6]. Consequently, this degeneration causes atrophy and weakening of the voluntary muscles in the limbs and trunk [7,8]. The clinical manifestations of SMA range from severe hypotonia and motor milestones failure in Type 1 to moderate motor difficulties in Type 4. Despite the motor neuron-centric view of SMA, recent insights have underscored its systemic nature, affecting multiple organ systems beyond the motor neurons. This broader impact likely stems from the ubiquitous expression of SMN protein and its varying levels affecting different tissues [1,4,9].

SMA is the most frequent genetic etiology of infant mortality and a significant cause of childhood morbidity, with a pan-ethnic incidence of 1/11.000 [1,10]. Varying incidence rates globally, estimated between 3.53 to 9.80 per 100,000 live births, with the highest rates in regions like Northeast Italy and West-Thuringen, Germany [9,11]. Racial disparities are noted, as seen in Cuba, where whites had an incidence of 8 per 100,000 compared to blacks at 0.89 per 100,000. The carrier state prevalence is around 1 in 54 [12], but its frequency varies significantly across racial groups, from 1 in 47 Caucasians to 1 in 72 African Americans [9]. Approximately 60% of SMA cases are classified as Type 1, with severe early symptoms. In comparison, Types 2 and 3 represent 27% and 12% of cases, respectively, showcasing a broader clinical spectrum based on SMN gene mutations and variations in genetic modifiers like SMN2 copy numbers [1].

The different forms of SMA are associated with numerous gene mutations and significant phenotypic variability [13]. Spinal muscular atrophy manifests in distinct forms, influenced primarily by the level of SMN protein produced due to a single-nucleotide polymorphism in the SMN2 gene, shown to be important in splicing. The severity, onset age, and SMA’s progression rate vary across its types. Type 1 SMA (also known as Werdnig–Hoffman disease), the most severe, affects infants before six months, resulting in significant motor function limitations and a survival rate of only 8% by 20 months, as these infants cannot sit independently. Type 2 SMA manifests after six months; children with this type achieve the ability to sit but cannot walk. Type 3 SMA typically appears in children and teenagers who initially can walk but experience progressive weakening and eventually lose walking ability. The rarest, Type 4 SMA, occurs in adults and features a prolonged progression of weakness in the lower extremities. Each type reflects differing outcomes based on the amount of functional SMN protein produced [1,5,12].

Quality of life (QoL) in SMA patients varies across domains, significantly impacting physical health and social functioning. SMA type, progression stages, and demographic factors like age and sex influence the complexity of assessing QoL. This variability challenges drawing definitive conclusions about the factors most affecting QoL in SMA patients [14,15]. Physical health is considerably compromised compared to mental health in SMA patients [12]. Issues such as item dependency, disordered thresholds, and multidimensionality may skew or misrepresent the patient’s actual state of health [14]. The evident disparity between physical and mental health scores calls for a balanced consideration of both aspects to truly understand and improve the holistic health of SMA patients. The findings underscore the necessity for comprehensive and tailored approaches in managing SMA, emphasizing the integration of physical and mental health interventions to enhance the overall well-being of affected individuals [4,13].

Nusinersen, risdiplam, and onasemnogene abeparvovec are currently the pivotal treatments approved to alter the progression of SMA [10,16]. These therapies enhance SMN protein production, which is indispensable for motor neuron function. Nusinersen and risdiplam target the SMN2 gene, modifying its splicing to increase the production of full-length SMN protein. Onasemnogene abeparvovec, on the other hand, involves a one-time intravenous delivery of a functional SMN1 gene using a viral vector, addressing the genetic cause of the disease.

Nusinersen, approved by the Food and Drug Administration in December 2016, was a significant breakthrough as it was the first drug to improve motor function and milestones in infantile and later-onset SMA [5,7,17,18]. This results in difficulties with movement, coordination, and the ability to perform everyday tasks, varying in severity based on the SMA type [11,19,20]. The clinical trials that prove the effectiveness of nusinersen in patients with SMA show an improvement in motor milestones and event-free survival [21]. Few studies evaluate motor skills improvement after nusinersen administration through specific scales [21,22,23,24], in which patients were not assessed in dynamics over more than 48 months during the treatment protocols. Risdiplam, approved in August 2020, offers ease of oral administration and has shown effectiveness across various SMA types, enhancing motor function and stabilizing disease progression as per multiple clinical trials [8,25]. Onasemnogene abeparvovec received FDA approval in May 2019 and has been transformative, particularly for infants with SMA Type 1, significantly improving survival and motor milestones compared to the natural progression of the disease [26].

The choice of therapy is influenced by various characteristics, including age, severity of disease, and specific SMA type, thereby necessitating a personalized approach to treatment. Integrating these therapies into clinical practice marks a pivotal era in SMA management, shifting the focus from symptomatic management to targeted genetic intervention [2,25].

This study aims to evaluate the influence of nusinersen on the motor skills of adult patients with SMA Type 2 and 3, using data collected for 54 months. The principal objectives are (1) assess the long-term impact of nusinersen on motor function over more than 4 years; (2) compare motor function between different SMA types using RULM and HFMSE scales; (3) identify demographic and genetic predictors that influence treatment outcomes and dropout rates; and (4) examine the correlation between SMA type, age at onset, and genetic background on treatment efficacy. In showing its effects on motor function, this research gives a more holistic view of the sustained effect of nusinersen and provides essential knowledge for optimizing treatment plans to improve care in patients with SMA.

## 2. Materials and Methods

We comprehensively assess the long-term efficacy of nusinersen and the progression of the disease over time in a cohort of adult Romanian patients diagnosed with SMA Type 2 and 3. By employing a longitudinal observational design and leveraging data collection methods, including anamnesis, detailed medical records, and validated motor function scales, the study sought to capture the nuanced impacts of the treatment over an extended period.

### 2.1. Study Design and Participants

We examined a cohort of 37 adult Romanian patients diagnosed with SMA, who were undergoing a 54-month therapeutic regimen of nusinersen between January 2019 and June 2023 in the Neurology Departments of “Fundeni” Clinical Institute in Bucharest and of CF Clinical Hospital in Timisoara, Romania.

The study meticulously collected and examined data through a multifaceted approach, which included anamnesis, detailed medical records, and evaluations using motor activity assessment scales, specifically the Hammersmith Functional Motor Scale Expanded (HFSME) and the Revised Upper Limb Module (RULM), previously validated for SMA [27].

The HFMSE was initially proposed for “high-functioning” patients with Type 2 and 3 SMA. This scale, comprising 33 items, assesses 13 clinically relevant domains from the Gross Motor Function Measure, including lying/rolling, crawling, crawling/kneeling, standing, and walking/running/jumping. Each item is scored from 0 to 2, with higher scores indicating the ability to perform the task. The total score ranges from 0 to 66, with lower scores indicating poorer motor function [28].

The RULM includes 20 items, with a maximum score of 37, where higher scores indicate better function. It assesses upper limb strength, precise movements, and abilities useful in everyday living [29].

The evaluation timeline was as follows: baseline, 6 months (after completing the first five doses of nusinersen as indicated by the protocol), and every 4 months thereafter.

The data encompassed several additional but critical variables extracted from the medical records that are pivotal in assessing the progression and response to the SMA treatment: the age at symptom onset, the specific type of SMA, the type of exon deletion, the number of copies of the SMN2 gene, and the scores from the assessment scales noted above. Each of these variables has a crucial role in understanding the nuances of the disease’s manifestation and the efficacy of nusinersen in altering the course of SMA in this particular population.

### 2.2. Statistical Consideration

This research applied models for survival analysis and mixed longitudinal models to test the durability and variability of the treatment’s effects across different types of SMA and along different timelines for improvement.

Continuous variables were summarized using medians and interquartile ranges, reflecting their distribution. Categorical variables were presented as frequencies and proportions.

In addition, we quantified the dropout rate, and we delved into the underlying reasons for why these patients chose to leave the study.

To compare the RULM and HFSME scores between groups, we used the Mann–Whitney U test for two classes and the Kruskal–Wallis test for more than two classes. We employed the Friedman test to compare the paired scores of the scales over different months. Additionally, we used the Wilcoxon signed-rank test to compare the paired test results between the RULM and HFSME scales.

For longitudinal data analysis, we used the Generalized Estimating Equation (GEE), a widely applied marginal model in clinical trials and biomedical studies for analyzing longitudinal or clustered data. GEE effectively predicted the average scores of the RULM and HFSME scales over time, accounting for the correlation between repeated measures within the same subjects and providing robust estimates of the effects of time and other predictors on the scores. By modeling the longitudinal data, we identified significant predictors that influence the progression of motor function in patients under treatment, thereby gaining insights into how these predictors affect the average scores of the scales over the study period.

The analysis was performed using R statistical software, version 4.4.1. Results are presented in graphic and tabular form, with statistical significance set at a *p*-value < 0.05 and a 95% confidence interval.

## 3. Results

This longitudinal study incorporated a range of demographic and genetic variables. It sought to delineate the patterns and predictors of treatment outcomes, thereby contributing valuable insights into the broader application of nusinersen in diverse patient settings.

### 3.1. Descriptive Analysis of Study Variables

The age at which patients began showing symptoms varies widely, with a median of 36 months. The range is broad, indicating significant variability in the age at onset, with the middle 50% of patients having an age at onset between 12 and 72 months. The median age when patients started treatment was 29 (range 18–70). The RULM scores also show variability, with a median score of 28. The interquartile range is narrower compared to age at onset (19–33), suggesting more consistency in RULM scores among patients. HFSME scores have a median of 13, with a relatively wide interquartile range (6–28), indicating variability in patient motor function scores. None of the variables follow a normal distribution (*p* < 0.05), suggesting that non-parametric statistical methods were used for further analysis of these variables. The results are presented in Table 1.

Most patients have SMA Type 3 (62.2%), while the others have SMA Type 2 (37.8%), showing that a higher proportion of patients in our study are affected by the later-onset, less severe form of SMA (Type 3). Most patients (91.9%) have deletions in Exons 7 and 8. Only a tiny percentage (8.1%) have a deletion in Exon 7 alone. This indicates that the most common genetic alteration in our cohort is the deletion of both exons. Most patients have three copies of the SMN2 gene (73.0%), a critical factor in the disease severity and response to treatment. A smaller proportion have two copies (24.3%), and very few have four copies (2.7%). Nine patients (24.3%) did not adhere to treatment, indicating a moderate dropout rate Twenty eight patients (75.7%) completed the study. The results are presented in Table 2.

### 3.2. Analysis of Treatment Dropout Rates

While studying patients with SMA undergoing treatment, it is crucial to understand the treatment dropout rates as they impact the overall effectiveness and interpretation of the outcomes.

The dropout rate (24.3%) for nusinersen treatment is primarily due to the difficulty of administration and refers to the fact that these patients discontinued the treatment with nusinersen and the associated evaluations.

To better understand the patients included in the study, we considered incorporating a longitudinal analysis of treatment adherence in our research. This approach allows us to track adherence patterns over time, providing valuable insights into patients’ long-term commitment to their treatment regimen and identifying any factors that may influence their adherence.

The 37 patients were enrolled in the study over a period of one and a half years, meaning not all of them began nusinersen therapy in January 2019. Patients who enrolled later in the study are currently ongoing in treatment. From Table 3, we can observe that in the 18th month of the study, one patient discontinued nusinersen therapy and switched to risdiplam. In the 26th month, two patients discontinued; one switched therapy and one patient due to pregnancy. In the 30th month, four patients discontinued; two by request, one due to the appearance of a lumbar hemangioma, and one due to a *Clostridium difficile* infection. In the 34th month of the study, one patient switched to risdiplam therapy and one patient discontinued without providing a reason.

Of the 9 patients who dropped out, five had SMA Type 3 and four had SMA Type 2. Regarding the genetic profile, seven patients presented with homozygous Exon 7 and 8 deletion, one with homozygous Exon 7 deletion and one with heterozygous Exon 7 and 8 deletion and a point mutation (c.821 C>T) in the Exon 7 of SMN1 gene. Six patients had three copies of SMN2, while the three remaining patients had two copies. Those results are similar to the patients who continued the treatment, as these genetic profiles are dominant.

### 3.3. Comparative Analysis of RULM and HFSME Scales

The Revised Upper Limb Module (RULM) scale was compared across different patient subgroups using the Mann–Whitney U and Kruskal–Wallis tests. The significant *p*-value (<0.001) indicates a statistically significant difference in the RULM scores between SMA Type 2 and SMA Type 3 patients. The median RULM score is higher for SMA Type 3 (38) than SMA Type 2 (22), suggesting that patients with SMA Type 3 generally have better upper limb function. Regarding exon deletion, the *p*-value (0.56) is insignificant, indicating no statistically significant difference in RULM scores between patients with Exon 7 deletion and those with Exon 7 and 8 deletions. The medians (30 vs. 27) are relatively close, suggesting similar upper limb function across these groups. The significant *p*-value (<0.001) indicates a statistically significant difference in RULM scores between those who dropped out of treatment and those who did not. The median RULM score is higher for patients who did drop out (34) than those who did not (27.5), indicating better upper limb function in patients who were followed up for a shorter period of time. The number of SMN2 copies resulted in a significant *p*-value (0.001), indicating a statistically significant difference in RULM scores among groups with different SMN2 copies. Patients with more additional copies of SMN2 tend to have higher median RULM scores (26 for two copies, 30 for three copies, and 31 for four copies), suggesting that a higher number of SMN2 copies is associated with better upper limb function. For the Mann–Whitney U test, degrees of freedom are not typically used as the test is non-parametric for SMA, exon deletion, and dropout status variables. Instead, we focus on the U statistic itself (the U statistic for SMA is 5072.5, the U statistic for exon deletion is 6061.50, and the U statistic for dropout status is 187). The Kruskal–Wallis test for number of copies SMN2 revealed two degrees of freedom and an H statistic of 12.80. The results are presented in Table 4.

The Hammersmith Functional Motor Scale Expanded (HFSME) scores were compared across different patient subgroups using the Mann–Whitney U and Kruskal–Wallis tests. Regarding SMA Type, the significant *p*-value (<0.001) indicates a statistically significant difference in HFSME scores between SMA Type 2 and SMA Type 3 patients. The median HFSME score is higher for SMA Type 3 (30) than SMA Type 2 (3), suggesting that patients with SMA Type 3 generally have better motor function as measured by the HFSME scale. In the case of exon deletion, the *p*-value (0.21) is insignificant, indicating no statistically significant difference in HFSME scores between patients with Exon 7 deletion and those with Exon 7 and 8 deletions. The medians (23 vs. 10) are distinct, suggesting that patients with a single Exon 7 deletion have and maintain better motor function compared to those with deletions of both Exons 7 and 8. Dropout status showed a non-significant *p*-value (0.3611), indicating that the differences between the HFSME scores of those who dropped out and those who did not were not statistically significant. The median HFSME score is higher for patients who did drop out (38) than those who did not (13), indicating better motor function in patients were followed up for a shorter period of time. The significant *p*-value (0.002) indicates a statistically significant difference in HFSME scores among groups with different numbers of SMN2 copies. Patients with more additional copies of SMN2 are inclined to have higher median HFSME scores (8 for two copies, 16.5 for three copies, and 23 for four copies), suggesting that a higher number of SMN2 copies is associated with better motor function. In the Mann–Whitney U test, degrees of freedom are not typically applicable as the test is non-parametric. Instead, the U statistic is the primary focus: for SMA, the U statistic is 54,320.00; for exon deletion, it is 6461.50; and for dropout status, it is 129. The Kruskal–Wallis test for the number of SMN2 copies revealed two degrees of freedom and an H statistic of 11.68. The results are presented in Table 5.

Due to varying dropout rates in the treatment data, we imputed the missing values with the median values calculated for each specific month to enable the use of the Friedman test. The significant *p*-values (<0.001) for RULM and HFSME scores indicate statistically significant differences across time points. This suggests that the scores on both scales change significantly over time. The results are presented in Table 6.

**Figure 1:**
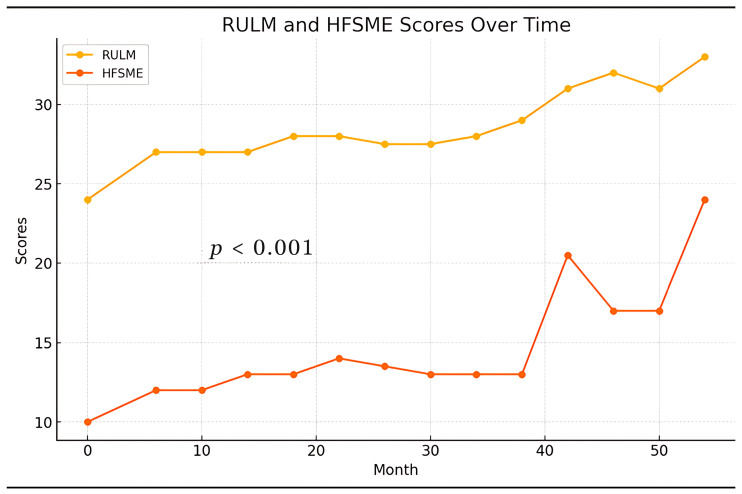
Paired measurements of RULM and HFSME scores over time.

The significant *p*-value (<0.001) indicates a statistically significant difference between the RULM and HFSME scores. This suggests that the measurements from the two scales are not equivalent, indicating that they may be capturing different aspects of motor function or responding differently over time. The results are presented in Table 6 and Figure 1.

### 3.4. Longitudinal Analysis of Motor Function for RULM and HFSME

To address the correlation among repeated measures, we employed Generalized Estimating Equations (GEEs) to analyze longitudinal data, providing both robust standard errors and more accurate and reliable estimates.

The GEEs analysis for RULM scores reveals significant predictors of upper limb function over time in patients under treatment. Specifically, time (months), SMA Type 3, older age at onset, and Exons 7 and 8 deletions are associated with higher RULM scores.

There is a statistically significant positive effect of time (measured in months) on RULM scores among patients under treatment. The RULM score increases by 0.05 points for each additional month, indicating a gradual improvement in upper limb function over time due to ongoing treatment. Patients with SMA Type 3 under treatment have significantly higher RULM scores than those with SMA Type 2. On average, the RULM score is 13.10 points higher for SMA Type 3 patients, suggesting better upper limb function in this group due to the treatment.

Age at onset has a statistically significant positive effect on RULM scores among patients under treatment. For each additional month in age at onset, the RULM score increases by 0.05 points. This indicates that patients who develop SMA symptoms later tend to have better upper limb function while receiving treatment. Patients with deletions in Exons 7 and 8 under treatment have significantly higher RULM scores than those with other exon deletions. On average, the RULM score is 6.12 points higher for patients with these deletions, indicating better upper limb function while under treatment. The results are presented in Table 7.

The GEEs analysis for HFSME scores reveals significant predictors of motor function over time in patients under treatment. Specifically, time (months), SMA Type 3, older age at onset, and deletions in Exons 7 and 8 are associated with higher HFSME scores. These findings suggest that these factors positively improve motor function performance among patients with SMA who are receiving treatment. There is a statistically significant positive effect of time (measured in months) on HFSME scores among patients under treatment.

For each additional month, the HFSME score increases by 0.08 points, indicating a gradual improvement in motor function over time due to ongoing treatment. Patients with SMA Type 3 under treatment have significantly higher HFSME scores than those with SMA Type 2. On average, the HFSME score is 16.43 points higher for SMA Type 3 patients, suggesting better motor function in this group due to the treatment. Age at onset has a statistically significant positive effect on HFSME scores among patients under treatment. For each additional month in age at onset, the HFSME score increases by 0.18 points. This indicates that patients who develop SMA symptoms later tend to have better motor function while receiving treatment. Patients with deletions in Exons 7 and 8 under treatment have significantly higher HFSME scores than those with other exon deletions. On average, the HFSME score is 12.31 points higher for patients with these deletions, indicating better motor function while under treatment. The results are presented in Table 8.

Summarizing, the longitudinal analysis of the study highlights significant variability in treatment outcomes among patients with SMA. The study found that patients with SMA Type 3 exhibited better retention and improved motor function than those with SMA Type 2. Genetic factors, such as the number of SMN2 gene copies and specific exon deletions, also significantly impacted patient outcomes. Patients with deletions in Exons 7 and 8 and those with more copies of the SMN2 gene demonstrated better upper limb and overall motor function. Longitudinal improvements in motor function, measured through RULM and HFSME scores, were significantly influenced by the time under treatment, SMA type, age at symptom onset, and specific genetic mutations, suggesting a nuanced interaction between these variables and treatment efficacy.

## 4. Discussion

This study uses real-world outcome data to provide critical insights into treating SMA with nusinersen, a targeted therapy that has transformed the management of this debilitating genetic disorder [7,17].

The long-term administration over 54 months in this cohort allowed for a thorough evaluation of the drug’s efficacy, impact on patient mobility and daily functioning, and dropout rates. An extensive literature review has identified several similar analyses on these variables in different SMA populations, but none covering such an extended period. Tscherter et al. describe the outcomes of 44 patients observed over 41 months [30], representing the most extended study found to date. Hjartarson et al. summarize results from five cohorts studied for a maximum period of 2 years [10], while Giess et al.‘s very recent review of the outcomes for patients with SMA treated with approved therapeutics offers an observational period of up to 48 months [31].

As per the 2018 recommendations, all SMA patients should benefit from regular (every 6 months) neurologic examinations using an appropriate functional scale and carried out by trained physiotherapists or physicians. The scale selection should consider the patient’s age, SMA type, and neurological status [32]. The most popular motor scales suitable for adults include Motor Function Measure 20 (MFM-20), Gross Motor Function Measure (GMFM), HFMSE, RHS (Revised Hammersmith Scale), 6-Minute Walk Test (6-MWT), RULM, Quantitative Muscle Testing (QMT), and Neuromuscular Gross Motor Outcome (GRO) [33]. HFMSE, RULM, and MFM are recommended for sitters, while 6-MWT, RULM, and HFMSE are considered the most relevant for walkers. The Revised Upper Limb Module [29,34] and the Hammersmith Functional Motor Scale Expanded [35,36] were pivotal in this research as they were found to be explicitly validated for SMA [27]. When used together, these scales provide an integrated approach to clinically evaluating SMA patients, offering quantitative measures that can be closely monitored over time to assess disease progression and treatment effectiveness [33,37].

The descriptive analysis, which included variables that predict the disease’s course and response to treatment, revealed some important characteristics of patients with SMA [1,38,39,40,41,42].

The variability in the age of SMA symptom onset indicates a complex interplay of genetic and possibly environmental factors [43]. This wide range from early to late childhood emphasizes the need for early surveillance and preemptive therapeutic strategies in a diverse pediatric population [44]. The median onset age of 36 months and the range of 12 to 72 months in our cohort align with the definition of SMA Type 3, highlighting the prevalence of later-onset and generally less severe manifestations of the disease [13].

This research builds on previous studies by emphasizing the involvement of genetic and phenotypic variability in influencing treatment outcomes [16,37,38,43]. The variability in exon deletions and SMN2 copy numbers among patients suggests that personalized approaches to SMA treatment might be necessary to optimize outcomes [44,45,46,47,48]. The study population mostly has deletions in Exons 7 and 8, suggesting a common genetic alteration and aiding in understanding nusinersen treatment effectiveness in this tailored genotype [49]. The presence of the hybrid gene (Exon 8 of SMN1 and Exons 1–7 of SMN2) is sporadic. These findings align with existing literature, suggesting that the three patients with this hybrid gene will likely exhibit a milder form of the disease [50,51,52]. Additionally, genetic data revealing that most patients have three copies of the SMN2 gene—a critical determinant of disease severity—further enriches this analysis, as the number of SMN2 copies is known to inversely correlate with disease severity for the intermediate phenotypes as Types 2 and 3 of SMA [53].

A good adherence to treatment was observed in our group, with 75.7% (28) of the total number (37) of patients continuing the administration of nusinersen after 54 months. However, the dropout rate of 24.3% raises concerns about treatment tolerability, the invasiveness of administration, and socioeconomic challenges associated with prolonged treatment These findings highlight the need for a more patient-centered approach, incorporating supportive therapies and addressing the broader needs of patients and their families [54]. A review of the literature on this subject offers heterogeneous results. Fox et al. reported an adherence to nusinersen treatment at 41% after 1 year and 39% after 2 years [55], similar to other groups as reported by Gauthier-Loiselle et al. [56], while Elman et al. [57] found that 67% of patients were still adherent after 24 months. Interestingly, Maggi et al. [24] reported a meager dropout rate of 2.5% over 6 months, likely due to the shorter study duration. The study ensured robust interpretations that reflected actual trends by focusing on statistically significant variables. SMA Type 3 patients had an apparently lower risk of treatment discontinuation than those with SMA Type 2, suggesting better adherence and possibly more effective management or tolerance of therapy in this subgroup, similar to findings by Gauthier-Loiselle et al. [56]. Future research should delve deeper into this subject, as our work is only the second to report a high dropout rate among Type 2 SMA patients. The study found no significant differences in dropout rates across various genetic profiles, indicating that psychosocial or healthcare-related factors—such as logistical challenges, increased dependency, concerns about treatment effectiveness, fear of side effects, poor prognosis, and communication issues—may significantly influence dropout rates. This supports the previously stated need for a holistic, personalized approach to patient care, incorporating medical and supportive interventions to improve treatment efficacy and quality of life [7,58,59].

The study suggests that nusinersen improves motor function, as enhancements in RULM and HFSME scores indicate. RULM analysis shows significant differences in upper limb functionality, with Type 3 patients performing better. Both Exon 7 deletions and more SMN2 copies were associated with better results. Patients who remained in the study showed continued motor function improvements and Type 3 patients had better motor function than Type 2 patients. There is a strong correlation between SMN2 copy numbers and motor function, highlighting the value of genetic testing for prognosis and treatment planning. Adherence to treatment did not appear to correlate with better motor function compared to dropouts when analyzing the medians scores of the two scales. The few patients who dropped out were followed for almost half the time compared to those who continued the treatment, which may explain these results. Additionally, the Mann-Whitney U test supported these findings by showing no significant differences. Other studies also emphasize the complexities of measuring functional improvements in SMA patients using RULM and HFSME scales [12,21,23,24,30,60].

While both scales showed similar general outcomes, a significant difference was observed in their ceiling effects. The RULM had a median score of 65% of the maximum at baseline, rising to 89% by the end of the surveillance period. In contrast, the HFSME had a median score of 15% at baseline, increasing only to 36%. The discrepancies in scores between the HFSME and RULM scales suggest that they capture different aspects of motor abilities or respond differently over time due to the nature of the functions they measure. These differences can be leveraged in clinical assessments to provide a more comprehensive understanding of a patient’s motor function across various dimensions. Additionally, limb-targeted therapies could yield widespread benefits [29,60,61,62,63,64]. Some studies also describe no significant benefit (but only RHS and 6MWT maintenance) in SMA Type 3 patients who started nusinersen after more than 18 years of age [65,66,67]. Regarding these observations, most of our patients were in this category with a median age of 29 when they started treatment but still showed significant improvement in motor milestones. More well-designed studies are welcomed to determine nusinersen’s effectiveness in SMA Type 3 when introduced in adulthood, taking into account also variables like “sitters” and “walkers” on the model of Maggi et al. [24].

The longitudinal analysis using Generalized Estimating Equations (GEEs) offers a robust framework for understanding motor function progression in SMA patients. This study is the first to apply GEEs analysis to an SMA cohort, which is a notable strength. GEEs effectively handles the complexities of repeated measurements, providing accurate estimates of treatment effects. The findings demonstrate consistent improvements in RULM and HFSME scores, supporting the long-term efficacy of nusinersen.

We acknowledge several limitations in our study. While the sample offers valuable insights into the efficacy of nusinersen for SMA Types 2 and 3, its representativeness is limited. With only around 250 genetically confirmed SMA cases in Romania and a predominance of Type 3 patients in the study, the findings may not be generalizable. Future research should include more diverse samples from multiple countries, covering different SMA types, genetic backgrounds, and regions, to better inform global SMA treatment strategies. The lack of a control group undergoing a placebo intervention, along with the small sample size, and the heterogeneous nature of the group also pose limitations, though these issues are common in real-world SMA studies [24,30,66,68]. The moderate dropout rate over time challenges the robustness of the study’s outcomes, representing a significant limitation in a small sample size such as ours. This attrition could either obscure potential benefits or skew the perceived efficacy of the treatment, particularly among less severely affected patients or those with specific genetic profiles [68,69,70]. Observing the cohort over such a long period can increase dropout risk due to various non-medical reasons. Other factors affecting motor deficiency, such as the deletion of the intact NAIP gene [71,72], are known to be significant but were not explored in our study due to financial and technical constraints. Future research, particularly randomized controlled trials, should investigate more thoroughly the incorporation of advanced genetic technologies and more diverse patient populations to understand the nuances and long-term benefits of nusinersen treatment fully.

In summary, this longitudinal study sheds light on the progression of SMA and the effects of nusinersen, highlighting its potential benefits across various genetic and demographic profiles as reflected in the improvements in RULM and HFSME scores. The findings could help refine patient selection criteria for nusinersen, optimize dosing schedules, and explore the potential for combining therapies based on individual genetic markers.

## 5. Conclusions

Conducted over 54 months, this study provides a valuable resource for ongoing studies and a foundation for future research to improve clinical outcomes for SMA patients globally. The long-term efficacy of nusinersen in adult SMA patients, especially those with Type 3, was strongly indicated. The findings revealed significant motor function improvements, with responses varying based on SMA type, age at onset, and genetic background. The Revised Upper Limb Module (RULM) and Hammersmith Functional Motor Scale Expanded (HFSME) effectively tracked these improvements. However, the study faced moderate dropout rates, highlighting the need for better patient retention strategies. Genetic factors, such as SMN2 copy number and exon deletions, were crucial in influencing outcomes, with patients having a later symptom onset generally showing better responses to treatment.

## Figures and Tables

**Table 1:** Analysis of numerical variables.

Variable	Median (Q25–Q75)	Shapiro–Wilk w	*p*-Value
Age at onset (months)	36 (12–72)	0.79	<0.001
RULM	28 (19–33)	0.88	<0.001
HFSME	13 (6–28)	0.80	<0.001

Abbreviations: RULM—Revised Upper Limb Module, HFMSE—Hammersmith Functional Motor Scale Expanded.

**Table 2:** Analysis of categorical variables.

Variable	Class	Frequency	Proportion
SMA Type	2	14	37.8%
	3	23	62.2%
Exon deletion	7	3	8.1%
	7 and 8	34	91.9%
Number copies SMN2	2	9	24.3%
	3	27	73.0%
	4	1	2.7%
Dropout status	Yes	9	24.3%
	No	28	75.7%

Abbreviations: SMA—Spinal Muscular Atrophy, SMN2—Survival Motor Neuron 2.

**Table 3:** Paired measurements of RULM and HFSME scores over time.

Month	Number of Patients in the Study	Patients Dropout
0	37	0
6	37	0
10	37	0
14	37	0
18	36	1
22	36	0
26	34	2
30	30	4
34	28	2
38	28	0
42	28	0
46	28	0
50	28	0
54	28	0

**Table 4:** Comparative analysis of RULM scores.

Variable	Class	Median	Mann–Whitney U/Kruskal–Wallis	Degrees of Freedom	*p*-Value
SMA	2	22	5072.50	-	<0.001
	3	38			
Exon deletion	7	30	6061.50	-	0.56
	7 and 8	27			
Dropout status	Yes	34	187	-	0.2437
	No	27.5			
Number copies SMN2	2	26	12.80	2	0.001
	3	30			
	4	31			

Abbreviations: SMA—Spinal Muscular Atrophy, SMN2—Survival Motor Neuron 2.

**Table 5:** Comparative analysis of HFSME scores.

Variable	Class	Median	Mann–Whitney U/Kruskal–Wallis	Degrees of Freedom	*p*-Value
SMA	2	3	4232.00	-	<0.001
	3	30			
Exon deletion	7	23	6461.50	-	0.21
	7 and 8	10			
Dropout status	Yes	38	129	-	0.3611
	No	13			
Number copies SMN2	2	8	11.68	2	0.002
	3	16.5			
	4	23			

Abbreviations: SMA—Spinal Muscular Atrophy, SMN2—Survival Motor Neuron 2.

**Table 6:** Paired measurements of RULM and HFSME scores over time.

Month	RULM		HFSME
0	24		10
6	27		12
10	27		12
14	27		13
18	28		13
22	28		14
26	27.50		13.50
30	27.50		13
34	28		13
38	29		13
42	31		20.50
46	32		17
50	31		17
54	33		24
Friedman	<0.001		<0.001
Wilcoxon		<0.001	

Abbreviations: RULM—Revised Upper Limb Module, HFSME—Hammersmith Functional Motor Scale Expanded.

**Table 7:** GEEs analysis results for predictors of RULM scores.

Predictor	Estimate	95% CI	*p*-Value
Month	0.05	0.02–0.08	0.001
SMA Type 3	13.10	5.73–20.48	<0.001
Age at onset	0.05	0.01–0.09	0.012
Exon 7 and 8 deletion	6.12	2.44–9.79	0.001

Abbreviations: SMA—Spinal Muscular Atrophy, CI—Confidence Interval, *p*-value—Wald test.

**Table 8:** GEEs analysis results for predictors of HFSME scores.

Predictor	Estimate	95% CI	*p*-Value
Month	0.08	0.05–0.12	<0.001
SMA Type 3	16.43	6.14–26.73	0.002
Age at onset	0.18	0.11–0.25	<0.001
Exon 7 and 8 deletion	12.31	5.86–18.76	<0.001

Abbreviations: SMA—Spinal Muscular Atrophy, CI—Confidence Interval, *p*-value—Wald test.

## Data Availability

The data presented in this study are available upon request from the corresponding author. Due to ethical restrictions, they are not publicly available.
